# DSD: A Discussion at the Crossroads of Medicine, Human Rights, and Politics

**DOI:** 10.3389/fped.2020.00125

**Published:** 2020-04-03

**Authors:** Petra De Sutter

**Affiliations:** Department for Reproductive Medicine, Ghent University, Ghent, Belgium

**Keywords:** DSD, intersex, human rights, Council of Europe, bio-ethics

## Abstract

Over the past years, the topic of “disorder/differences in sex development (DSD)” or “intersex” people has become subject of the international political agenda. In 2017, a resolution of the Parliamentary Assembly of the Council of Europe argued that the practice of surgically modifying intersex children's genitals without medical necessity and without consent of the person concerned is a human rights violation. This resolution and related statements might impact heavily on pediatric urologists and their practice. While this resolution concerns a form of soft law and is not directly enforceable in member states, it might impact the national debates concerning legislation and medical guidelines on DSD. Consequently, this article reflects on this discussion by elaborating on the importance of human rights in our evolving understanding and legislation on DSD and other gender and sexuality issues in general. It constitutes a plea for a dialogue between medical professionals, lawmakers and human rights scholars which would lead to legislation and medical guidelines that take a holistic and rights-based approach.

## Introduction

The complex dossier of “disorder/differences in sex development (DSD)” or “intersex” people^1^ has been subject to several political debates in the last decade. For a long time, the situation of intersex people has been treated as a purely medical issue. Already in the 1990s this approach was criticized by activists, who claimed that the practice of surgically modifying intersex children's genitals without medical indication and without the consent of the person concerned is a human rights violation. Following advocacy efforts, the topic has been subject of several political debates at global level since the early 2010s.

At European level, an important resolution has been the 2017 resolution from the Parliamentary Assembly of the Council of Europe on “promoting the human rights of and eliminating discrimination against intersex people” (1). The resolution strongly condemns the medically unnecessary, “sex- normalizing surgery” on intersex babies. It was furthermore stated that intersex people should be offered health care by a specialized, multidisciplinary team taking a patient-centered and holistic approach. The Assembly also said that intersex people should have access to legal recognition of their gender identity and that governments should raise public awareness of the rights of intersex people to ensure their full acceptance, without stigmatization or discrimination. In addition, it was stressed that there is a need to collect more data and carry out further research into the situation and rights of intersex people, including into the long-term impact of surgery and other treatments.

This resolution and related statements might impact heavily on pediatric urologists and their practice. The resolution is a form of soft law and cannot be directly enforced in national practice. However, the recommendation can play an important role in national debates on medical guidelines and could eventually lead to hard laws in individual nations. Consequently, a debate is needed between all concerned parties. To contribute to this debate among pediatric urologists, I was invited in 2018 at the 29th ESPU Congress to provide a lecture on this topic, as I was a member of the Parliamentary Assembly of the Council of Europe at the time the resolution on intersex people was being debated. This article is based on the speech that I provided at this Congress^2^.

Within this article, I will discuss some basis aspects of human rights law and explain why human rights are involved in the discussion on intersex people. I will highlight the importance of human rights to protect minorities, explain why human rights thinking sometimes clashes with bio-ethical frameworks, and discuss how the discussions on intersex people are part of a wider discussion on evolving understanding and legislation on gender and sexuality issues. In the discussion, I plead for a dialogue in all countries between medical professions, lawmakers and human rights scholars, which would lead to legislation and medical guidelines that take a holistic and rights-based approach on the matter.

## Human Rights: Protecting Minorities

The concept of human rights dates back to the age of the Enlightenment (2). John Locke supported the idea of natural rights upon which the government may not infringe and later Jean-Jacques Rousseau turned these ideas into the idea of a “social contract.” These ideas culminated in both the American declaration of independence in 1776 and the French Declaration of the Rights of Man and the Citizen in 1789. Our modern human rights views date back from the period after the Second World War. Following the horrors of the war, it was recognized that human rights were needed to protect minorities. Consequently, international agreements were developed, which resulted in 1948 in the Human Rights Declaration of the UN and the European Convention for the Protection of Human Rights and Fundamental Freedoms of the Council of Europe in 1950.

The underlying principle of the concept of human rights is the idea that people have inherent rights simply because they are human. Human rights give fundamental protection to individuals to allow equal participation in democracies. They protect citizens against possible excesses of democracy by focusing on the disadvantaged and marginalized population. By taking this minority approach, they are protecting society from what Alexis De Tocqueville called the “tyranny of the majority.” Human rights can be moral rights but can also be legal rights if they are inscribed in laws in member states. For example, the European Convention can be enforced at the European Court of Human Rights.

## Deontologists vs. Utilitarians

The philosophical basis for the protection of human rights is linked to the moral philosophy of Emmanuel Kant. He endorsed the ideals of equality and moral autonomy and he ascribed these ideals to the capacity for rational thinking of human beings. As human beings that can think for ourselves, we can also give rights to ourselves. These human rights do not depend on some divine power, but we can come to them through reasoning. Central to the moral theory of Kant is the idea of a “categorical imperative,” which means that you should act as you would want all other people to act toward all other people. This also implies that human rights are universal, absolute and unconditional. Kant's thinking is an example of the so-called deontological moral theory, according to which an action should be based on whether that action itself is right or wrong under a series of rules, rather than based on the consequences of the action.

However, even at the start, the concept of human rights was criticized by philosophers such as Jeremy Bentham and John Stuart Mill, who belong to the so-called utilitarian philosophical school. This school believes that the state should do what is necessary to obtain the maximum benefit for the maximum of people. According to this thinking, it is the consequence of what you do that is important, for the majority of the people. This clashes with the concept of individualized human rights, which certainly not always maximize utility.

## Human Rights and Bio-Ethics

The clash between the so-called deontologists and the utilitarian school is also relevant for medical practice, as contemporary bio-ethics can be considered a compromise between philosophers of the two schools. This can be illustrated by looking at the four central principles of medical ethics being autonomy, justice, non-maleficence and beneficence. Only the two first principles can go back to both Kantian and utilitarian philosophy. Non-maleficence goes back to Hippocrates and beneficence is unique to biomedical ethics and cannot be easily linked to human rights theory.

There are two fundamental differences between human rights and medical ethics. One is that the focus of human rights is on the state level action, while medical ethics are focused on a person-to-person- relationship. The concept of human rights is a social, political instrument whereas medical ethics determine how doctors deal with patients. The second difference relates to the concept of benevolence, which is central in medical ethics to decide how patients should be treated. But this concept cannot be linked to human rights discourse, where there is no place for empathy. You do not get rights because somebody likes you or somebody decides to do the best for you. You get rights because they are absolute and non-negotiable, because they are the basis of how our political systems work in democracies. So of course human rights and medical ethics can be parallel mechanisms which can both help to do the best for the patients, but sometimes the two will conflict with each other.

The different way of thinking between human rights and medical practice can also be illustrated by looking at the concept of evidence-based medicine, which is in essence a utilitarian concept. Evidence- based medicine is using statistics to show what is the best for the majority of patients. Scientists are not looking at every individual patient who could suffer from one or another complication nor do they monitor whether their rights have been infringed upon.

The increased importance attached to human rights has led to an increased focus on protecting minorities in medical discussions. For example, the rights of people with disabilities and LGBTI people are being better protected today than decades ago. Another concrete example is the discussion on donor anonymity, on which I have written a report in the Parliamentary Assembly of the Council of Europe (3). About 30 years ago when we started with sperm donations, doctors were instructed that the identity of the donor was of nobody's concern and that people should keep it secret. Today, we are confronted with donor children, who state that their rights have been violated, because for instance the convention of the rights of the children by the UN states that the child should have the possibility to know its origins. Consequently, many of these donor children have become activists and have been trying to change the law and abolish anonymity. Many human rights experts would agree with them, because from a human rights perspective if there is a minority of children whose rights have been infringed upon, you should change the law and abolish anonymity.

## Evolution From Disorder To Variation

Throughout the debate in the Council of Europe on the human rights of intersex people, a lot of discussions took place on terminology. Decades ago, medical students were being taught by terms such as “pseudo hermaphroditism.” As these terms were thought to be very stigmatizing, they were replaced by the term “intersex.” This term has been preferred by human rights activists and it has become a sort of “identify” defining term. To be intersex becomes something to be proud of, rather than a disease. The exact same evolution took place for sexual orientation, where heteronormativity was challenged by homosexuality, which first was seen as a disease, a disorder, and now is seen as a variation, which does not need any treatment or correction.

However, the term “intersex” was also criticized because intersexuality has nothing to do with sexuality and because the term is poorly defined. Consequently, since the Chicago Consensus statement of 2006, medical professions have mostly adopted the term “disorders of sex development” (DSD), which refers to a group of distinct congenital conditions in which development of chromosal, gonadal or anatomical sex is atypical. Yet, many intersex activists rejected the DSD-terminology, notably because the term “disorders” may imply that their bodies have a problem that needs to be “fixed.” Some have sought to find more neutral language, and suggested the terms “differences in sex development” or “variations in sex characteristics.”

These discussions on terminology are important as terminology creates reality. Moreover, these discussions are not unique to the topic of intersex people. In many subjects dealing with gender and sexuality there has been an evolution in terminology from “disorder” to “variation,” reflecting the evolving societal understanding on these issues. For example, homosexuality was considered, until 1973, to be a mental disorder. The Diagnostic and Statistical Manual of Mental Disorders (DSM) used the term “sexual orientation disturbance.” In 1973, it was changed and now it is considered a variation of sexual orientation. While in the past we used to have conversion therapies for homosexuals, we are talking now about equal rights for people of all sexual orientations. When it comes to gender identity, the terminology has changed from sex change to gender reassignment to gender confirmation. In addition, there has been an evolution in many societies where gender is not considered binary anymore. Instead of the male/female-distinction, a lot of non-binary gender forms are currently being accepted.

In line with the abovementioned examples, the discussion on intersex people is subject to an inevitable evolution from a compulsory medical treatment toward self-determination and informed consent-based, autonomous decisions. This change in terminology and thinking is entirely conform with a human rights approach.

## Reflections on Patient- and-Family-Centered Care

In an open letter to the Council of Europe, Katja Wolffenbuttel and Piet Hoebeke from the European Society for Pediatric Urology complimented the authorities that complied the report and recognized the resolution as a solid starting base for a dialogue on the topic (4). However, the authors did not agree with the statement that surgical intervention in children with DSD should only be applied in emergency conditions, as it is discordant with the broad definition of health of the WHO. Furthermore, they highlighted that “parents implicitly act in the best interest of their children and should be respected as their outstanding representatives, and should not be put aside by claiming prohibition regulations regarding the well-informed decisions they make on their behalf.” Consequently, they proposed to extend the concept of “multidisciplinary patient-centered care” to “multidisciplinary patient-and family- centered care.”

Of course it is important that parents are part of the decision making process, as they bear a lot of responsibility for what is best for their children. However, in a lot of human rights discussions it can be asked whether parents always are going for the best interest of their child. Sometimes states have to intervene because parents are not doing the best thing for their child. For example, when it comes to discussions on sexual education, there is a discussion in international for a whether this should be a parents responsibility or whether this should be a state responsibility as well as some parents might not provide the full, correct information to their children. There is also the discussion on female genital mutilation. While this is a severe human rights violation, parents still prefer to get their daughters cut. Both examples illustrate that parents are not always doing what is best for their children, even if they believe they do. If we are honest, we should even acknowledge that doctors are no always doing what is best for their patients. Therefore, a human rights perspective is very important to protect individuals.

## Discussion

As mentioned in the introduction, the resolution of the Council of Europe is not legally binding, but could influence the debate on creating national legislation on the topic in several European states. In Malta, legislation already exists since 2015. The “Gender identity, Gender Expression, and Sex Characteristics Act” forbids sex assignment treatments and/or surgical intervention to the sex characteristics of a child which can be deferred until the person to be treated can provide informed consent, unless in exceptional circumstances (5). In 2018, Portugal became the second nation in the world to ban medically unnecessary surgery on the genitals of intersex infants. In February 2019, the European Parliament adopted a resolution in which it strongly condemns sex-normalizing treatments and surgery and encourages Member States to adopt legislation on it. So it is only a matter of time before these discussions take place in several countries.

In case these debates take place, I plead for a constructive dialogue between medical experts, lawmakers and human rights thinkers. It is crucial to take a holistic view. One should not only look at the important medical concerns, but also fully understand the full range of human rights issues affecting intersex people, including the right to physical integrity and questions around informed consent. Human rights are universal; their enjoyment must never depend on the sex characteristics of the person. Most importantly, medical practitioners and human rights activists should understand that they talk a different language and that their approach about what is best sometimes differs. Mutual understanding can hopefully lead to guidelines and legislation which better combine both the biomedical ethical views and the human rights approach. In addition, it is important to collect data and evidence as much as possible, whatever practice is performed or legally enforced. Only then will it be possible to compare different approaches and work toward evidence-based policy making.

I plead for an open and not a defensive attitude on behalf of the medical community. If medical experts, human rights activists and politicians do not speak the same language and do not understand each other's arguments, the dialogue will be difficult. It is to be hoped that pediatricians will be heard by politicians, so that possible legislation will take into consideration their arguments as well as the arguments of activists, in order to create legislation that leads to the best possible outcome for people with DSD.

## Author's Note

PD is a Member of the European Parliament for the Greens/EFA and a former senator and former member of the Parliamentary Assembly of the Council of Europe.

## Author Contributions

The author confirms being the sole contributor of this work and has approved it for publication.

### Conflict of Interest

The author declares that the research was conducted in the absence of any commercial or financial relationships that could be construed as a potential conflict of interest.

## Footnotes

^1^As will be discussed in this paper, there is debate about the correct terminology. While medical professionals prefer the term “DSD,” some activists prefer the term “intersex” and the latter term is also most commonly used at the political level. In this article, both terms will be used interchangeably.

^2^A recording of this lecture can be viewed here: https://www.espu.org/members/video-broadcast/319-lecture-dsd-at-the-crossroads-of-medicine-human-rights-and-politics-at-helsinki-congress-2018.
